# Concordance of programmed death-ligand 1 expression between primary and metastatic non-small cell lung cancer by immunohistochemistry and RNA *in situ* hybridization

**DOI:** 10.18632/oncotarget.20254

**Published:** 2017-08-14

**Authors:** Hye Ryun Kim, Yoon Jin Cha, Min Hee Hong, Manoj Gandhi, Shauna Levinson, Inkyung Jung, Jin Gu Lee, Chang Young Lee, Byoung Chul Cho, Sang-Jun Ha, Hyo Sup Shim

**Affiliations:** ^1^ Yonsei Cancer Center, Division of Medical Oncology, Yonsei University College of Medicine, Seoul, Korea; ^2^ Department of Pathology, Yonsei University College of Medicine, Seoul, Korea; ^3^ Thermo Fisher Scientific 3450 Central Expressway, Santa Clara, CA, USA; ^4^ Department of Biostatistics and Medical Informatics, Yonsei University College of Medicine, Seoul, Korea; ^5^ Department of Thoracic and Cardiovascular Surgery, Yonsei University College of Medicine, Seoul, Korea; ^6^ JE-UK Institute for Cancer Research, JEUK Co., Ltd., Gumi-City, Kyungbuk, Korea; ^7^ Department of Biochemistry, College of Life Science & Biotechnology, Yonsei University, Seoul, Korea

**Keywords:** programmed death ligand-1, non-small cell lung cancer, immunohistochemistry, RNA *in situ* hybridization

## Abstract

We investigated the concordance of programmed death-ligand 1 (PD-L1) expression between primary cancer at initial diagnosis and metastasis at recurrence in resected non-small cell lung cancer (NSCLC). PD-L1 expression was evaluated using the SP142 assay in 37 NSCLC patients with paired primary lung cancer and surgically resected metastases at recurrence. PD-L1 positivity was defined as immunohistochemistry (IHC) and also evaluated by RNA *in situ* hybridization (RISH). The concordance rate of PD-L1 between primaries and metastases and correlation with clinicopathological factors were analyzed. PD-L1 expression was higher in squamous cell carcinoma, wild-type *EGFR*, and smokers than in non-squamous carcinoma, mutant *EGFR*, and never smokers, respectively. PD-L1 positivity was observed in 18.9% of primaries and 21.6% of metastases. IHC demonstrated 78.4% concordance of PD-L1 positivity between primary and metastatic cancers. In 10.8% of cases, PD-L1 positivity was higher in primaries than in metastases, and vice versa in the remaining 10.8%. By PD-L1 RISH, 35.1% of primaries and 27.0% of metastases demonstrated PD-L1 positivity. There was 62.2% concordance in PD-L1 by RISH between the primaries and metastases. Our results thus highlight the clinical importance of replacing metastases with primary archival tissue, particularly when re-biopsy is difficult at recurrence.

## INTRODUCTION

Non-small cell lung cancers (NSCLCs) comprise nearly 80% of lung cancers and have a dismal prognosis, with an overall 5-year survival rate of only 15% [1]. The treatment landscape of NSCLC has transformed with the approval of programmed death-1 (PD-1) and programmed death ligand-1 (PD-L1) blockade agents such as nivolumab, pembrolizumab, atezolizumab, and pembrolizumab in patients with ≥50% PD-L1 expression as a front line of therapy [2–7]. PD-1 is expressed on the surface of activated T cells [8]. Tumor cells and immune cells in the tumor microenvironment can express PD-L1 and PD-L2 [8, 9]. PD-L1 interaction with PD-1 on T cells can induce the downregulation of T-cell function and enable tumor growth and persistence through immune evasion [8]. These immune checkpoint blockades have shown remarkable antitumor activity and long-duration response across a wide range of cancer types, including NSCLC [8].

Currently, PD-L1 expression evaluated by immunohistochemistry (IHC) of formalin-fixed paraffin-embedded (FFPE) tissue samples is used as a companion diagnostic test to predict the response to PD-1 blockade [2–5, 10]. Treatment outcomes of PD-1 blockades are better in patients whose tumors express higher levels of PD-L1 than in those expressing low or no PD-L1 [11]. Knowledge of PD-L1 status can guide treatment decisions; therefore, assays reliably and accurately measuring tumor PD-L1 expression levels are warranted [11]. PD-L1 expression levels in tumor cells might not be consistent, because they can be altered by IFN-γ secretion or constitutive oncogene activation [12–15]. Moreover, chemotherapy or radiotherapy can affect PD-L1 levels of tumor cells and immune cells [12]. Thus, PDL-1 expression levels can differ between primary lung cancer at first diagnosis and metastatic cancer at recurrence [9, 16, 17]. However, data on the correspondence between PDL-1 expression levels at primary lung cancer and metastatic sites are limited. Determining whether PDL-1 expression in archival primary lung cancer is maintained in a metastatic site is very important in clinical decision-making in terms of PD-1 pathway inhibitor therapy. To date, no studies have evaluated and compared PD-L1 expression between primary lung cancer and metastatic tumor sites.

PD-L1 positivity has been found to be a predictive factor for non-squamous histology but not for squamous cell histology [2, 3]. Although PD-L1 expression is currently a predictive biomarker for PD-1 blockade, the prediction accuracy is not high enough to confirm the drug efficacy. The use of PD-L1 IHC to predict response is affected by constraints such as intratumoral PD-L1 heterogeneity, PD-L1 expression, IHC platforms, detection antibodies, and positivity cut-off level [9, 18]. Examining PD-L1 status using RNA *in situ* hybridization (RISH) might circumvent these limitations [10, 19, 20]. Velcheti *et al*. recently demonstrated that PD-L1 mRNA expression above the detection threshold showed statistically better outcomes by RISH than by IHC in two cohorts of NSCLC patients [10]. The visualization of RISH facilitates accurate determination of the cellular location of tumor cells or infiltrating immune cells [10, 19]. Such a technique is more accurate than qPCR and is amenable to machine learning image analysis.

Here, we aimed to investigate the concordance of PD-L1 level between archival primary tumors at initial diagnosis and metastasis at recurrence in surgically resected NSCLC by IHC and RISH.

## RESULTS

### Clinicopathological data

The baseline demographics of the 37 patients analyzed are summarized in Table 1. The median age of patients at diagnosis was 63 years (range, 33–77 years); most of the patients were men (73.0%) and current or ex-smokers (70.3%). Most cases (27/37, 73.0%) showed adenocarcinoma histology. The proportions of stages at the initial diagnosis were 56.7% stage I, 24.3% stage II, and 18.9% stage III. Among 27 adenocarcinoma patients, eight harbored *EGFR* mutations, including L858R at Exon21 (n = 3), Exon19 deletion (n = 4), and Exon 20 insertion (n = 1). The remaining 29 patients (78.3%) harbored wild-type *EGFR*. The sites of resected metastatic tumor were the lung (20/37, 54.1%), brain (4/37, 10.8%), pleura (5/37, 13.5%), and others (8/37, 21.6%). The median time from initial diagnosis to recurrence was 17.8 months (range, 2.5–52.5).

**Table 1:** Baseline characteristics

Characteristics	Total (n=37)n (%)
*Age, year*	
Median (Range)	63 (33-77)
*Sex*	
Male	27 (73.0)
Female	10 (27.0)
*Metastatic sites*	
Lung	20 (54.1)
Pleural	5 (13.5)
Brain	4 (10.8)
Others	8 (21.6)
Pathology	
Squamous cell carcinoma	10 (27.0)
Adenocarcinoma	27 (73.0)
Smoking History	
Never smoker	11 (29.7)
Ex and current smoker	26 (70.3)
*AJCC7 stage*	
Stage I	21 (56.7)
Stage II	9 (24.3)
Stage III	7 (18.9)
EGFR mutation status	
Exon 19 del	4 (10.8)
Exon21 L858R	3 (8.1)
Exon20 insertion	1 (2.7)
Adjuvant chemotherapy	
Yes	21 (56.8)
Surgical biopsy method	
Wedge resection of lung	16 (43.2)
Lobectomy of lung	3 (8.1)
Excision	14 (37.8)
Craniotomy	4 (10.8)

### PD-L1 expression by IHC in primaries and metastases

The IHC method with 5% cut-off points showed 18.9% (7/32) of primaries and 21.6% (8/37) of metastases to be PD-L1 positive. PD-L1 expression was found to be correlated with clinicopathological features. In primary tumors, higher PD-L1 positivity was observed in squamous cell carcinoma (SCC) and wild-type *EGFR* than in adenocarcinoma and mutant *EGFR*, respectively (adenocarcinoma, 13.5% vs. 5.4%, *P* = 0.005; mutant *EGFR*, 18.9% vs. 0% *P* = 0.05; Table 2). These findings were consistent in both primaries and metastases. Current or ex-smoker status tended to increase PD-L1 positivity in primary sites (Table 2). Besides these findings, there was no significant difference in PD-L1 expression across clinical factors (stage, age, sex, and site of metastasis) between primaries and metastases. Furthermore, patients with *EGFR* mutations had 0% and 2.7% PD-L1 positivity in primaries and metastases, respectively (Table 2).

**Table 2:** Association of PD-L1 of primary and metastasis with clinical factor

	Primary PD-L1 IHC		P-value	Metastatic PD-L1 IHC		P-value	Primary PD-L1 ISH		P-value	Metastatic PD-L1 ISH		P-value
	positive	negative		positive	negative		positive	negative		positive	negative	
**Histology**			***0.005***			***0.012***			0.691			0.463
Adenocarcinoma	2 (5.4%)	26 (70.3%)		3 (8.1%)	25 (67.6%)		9 (24.3%)	19 (51.4%)		7 (18.9%)	21 (56.8%)	
Squamous carcinoma	5 (13.5%)	4 (10.8%)		5 (13.5%)	4 (10.8%)		4 (10.8%)	5 (13.5%)		3 (8.1%)	6 (16.2%)	
**EGFR mutation**			***0.056***			***0.038***			0.711			0.832
Mutant	0 (0%)	11 (29.7%)		0 (0%)	11 (29.7%)		3 (8.1%)	8 (21.6%)		3 (8.1%)	8 (21.6%)	
Wild type	7 (18.9%)	19 (51.4%)		8 (21.6%)	18 (48.6%)		10 (27.0%)	16 (43.2%)		7 (18.9%)	19 (51.4%)	
**Smoking history**			***0.064***			0.672			0.602			0.639
Current/ex smoker	7 (18.9%)	19 (51.4%)		5 (13.5%)	21 (56.8%)		9 (24.3%)	17 (45.9%)		7 (18.9%)	19 (51.4%)	
Never smoker	0 (0%)	11 (29.7%)		3 (8.1%)	8 (21.6%)		4 (10.8%)	7 (18.9%)		3 (8.1%)	8 (21.6%)	

**Figure 1:**
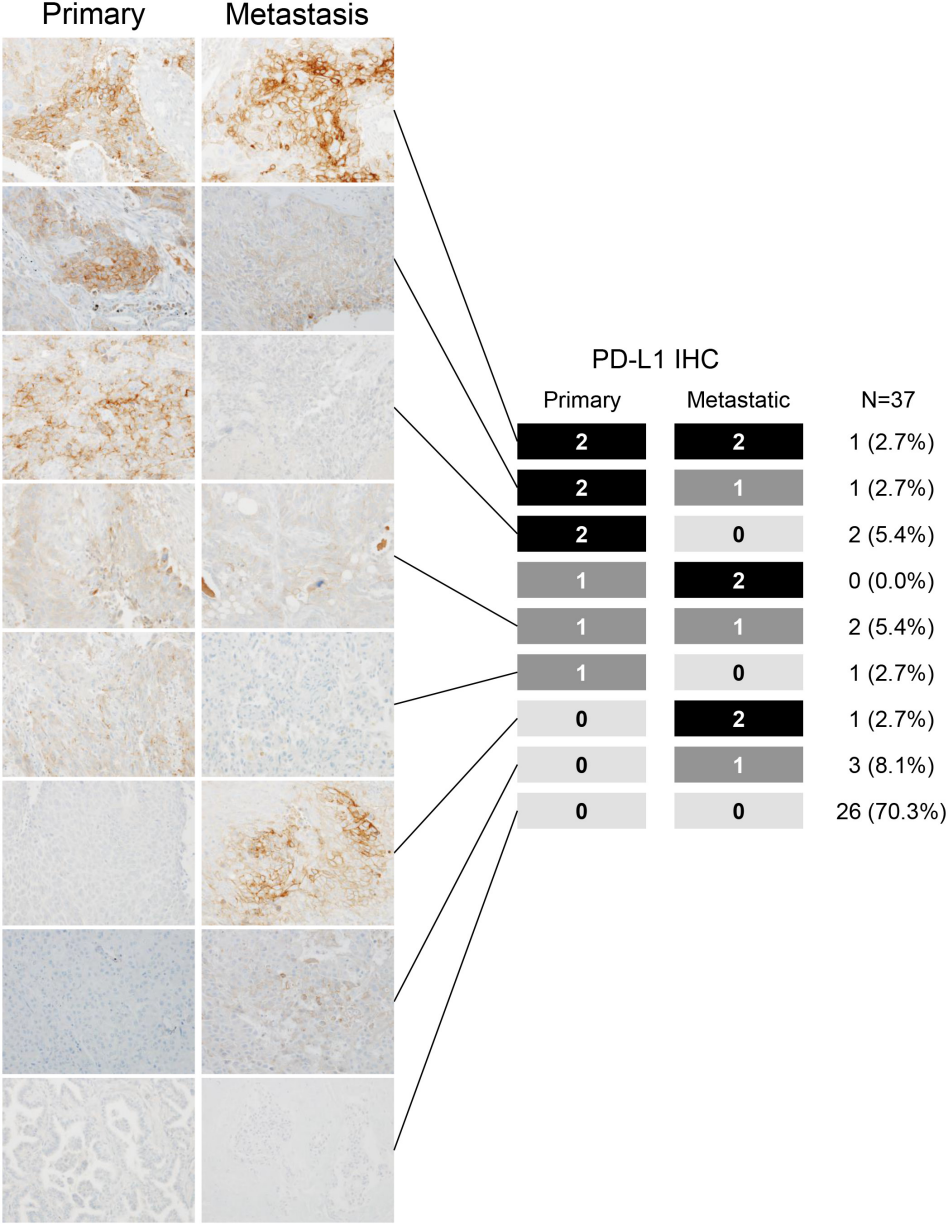
Programmed death-ligand 1 (PD-L1) expression levels in paired surgically resected primary and metastatic tissues with representative PD-L1 immunohistochemistry images Most cases showed concordance of PD-L1 immunohistochemical (IHC) staining between primary and metastatic sites.

There was 78.4% concordance of PD-L1 IHC staining between primary and metastatic sites (Cohen's κ coefficient = 0.374, 95% CI 0.055–0.693, *P* = 0.02); in 10.8% cases, the PD-L1 score of primaries was higher than that of metastases, and in the remaining 10.8%, it was the other way around (Figure 1, Supplementary Figure 1). Thus, PD-L1 status in primary NSCLC can predict the PD-L1 status of metastases in almost 80% of cases. Semi-quantitative analyses demonstrated that the H-score of PD-L1 in primaries corresponded with that in metastases (*P* = 0.33, Supplementary Figure 2). These semi-quantitative data demonstrated that PD-L1 expression in most cases is consistent between primaries and metastases.

### PD-L1 by RISH in primary and metastatic sites

**Figure 2:**
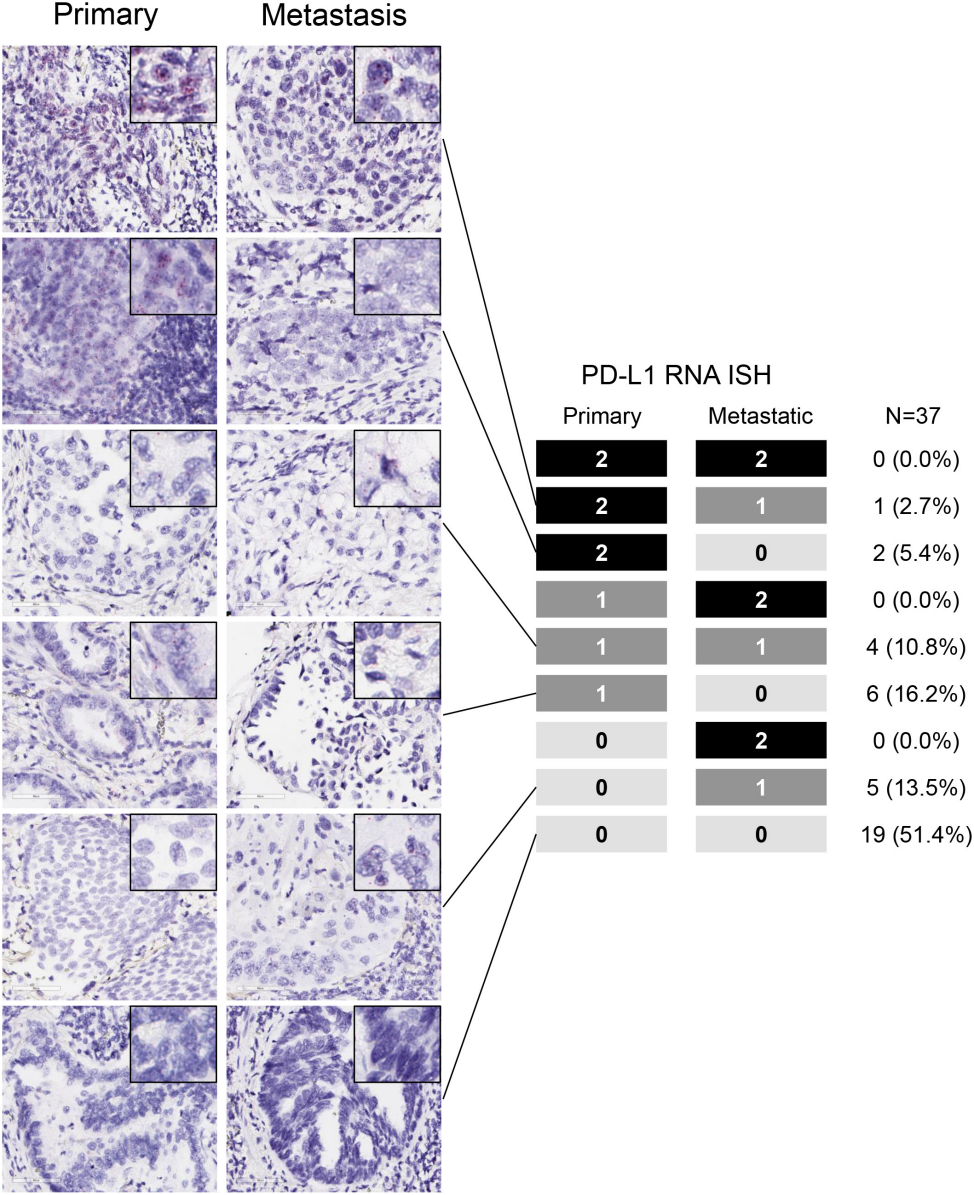
PD-L1 mRNA levels in paired surgically resected primary and metastatic tissues with representative PD-L1 RNA *in situ* hybridization (RISH) images Most patients demonstrated concordance of PD-L1 RISH between primary and metastatic sites.

According to PD-L1 RISH, 35.1% (13/37) of primaries and 27.0% (10/37) of metastases were PD-L1 positive. PD-L1 RISH positivity tended to be high in SCC but low in never/light smokers or individuals with *EGFR* mutation. There was 62.2% concordance in PD-L1 RISH between primaries and metastases (Cohen's κ coefficient = 0.186, 95% CI −0.139–0.325, *P* = 0.249); in 24.3% of cases, the PD-L1 score of primaries was higher than that of metastases, and vice versa in 13.5% cases (Figure 2, Supplementary Figure 1). We also evaluated the concordance across the two different platforms, including IHC by SP142 and RISH. Between the two different platforms, 66.7% of primaries and 73.0% of metastases showed agreement.

### Survival outcomes in relation with PD-L1 in primaries and metastases

**Figure 3:**
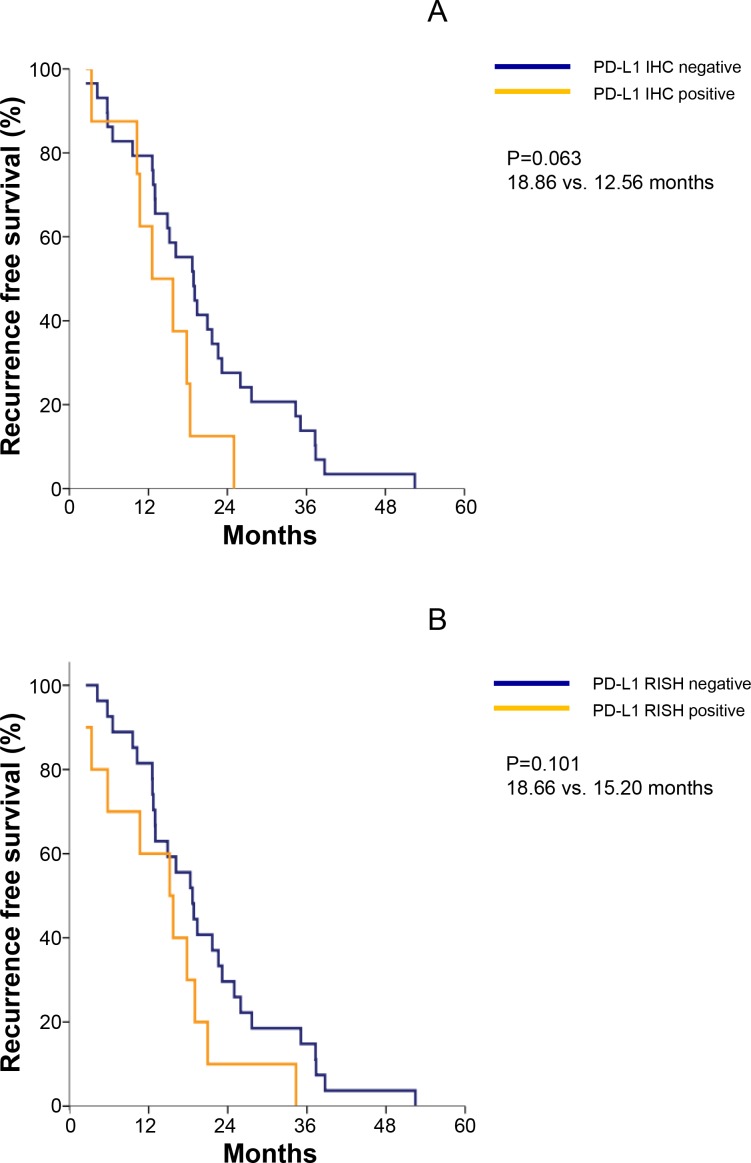
**(A)** Median recurrence-free survival (RFS) was 18.86 months for PD-L1 IHC-negative and 12.56 months for PD-L1 IHC-highly positive samples. **(B)** Median RFS was 18.66 months for PD-L1 RISH-positive and 15.20 months for PD-L1 RISH-negative.

We evaluated the correlation of PD-L1 expression by IHC and RISH with the survival outcomes in 37 patients. Patients without PD-L1 IHC of metastatic lesion tended to demonstrate improvement of RFS (18.8 *vs.* 12.5 months, *P* = 0.06) and those without PD-L1 RISH of metastasis showed numerical improvement of RFS (18.6 *vs.* 15.2 months, *P* = 0.10; Figure 3). PD-L1 IHC or RISH of primary tumor samples was not significantly associated with RFS and OS.

## DISCUSSION

High PD-L1 expression is used as a biomarker to predict the benefit from treatment with PD-1 signaling pathway inhibitors in NSCLC [16, 18]. Testing PD-L1 expression in tumor tissues is mandatory to identify patients most likely to respond to anti-PD-1 blockades [11]. Therefore, the use of archival primary tumor tissue to assess PD-L1 status might help guide decisions regarding PD-1 blockade treatment when patients experience recurrence after surgery. We conducted this study to determine whether PD-L1 expression in primary lung cancer is concordant with that in metastatic lung cancer when recurrence occurs in NSCLC patients who have undergone curative resection. Importantly, almost 80% of cases demonstrated consistent PD-L1 levels between primaries and metastases by PD-L1 IHC. This relatively high rate of concordance between primaries and metastases provides evidence that primary archival tissue can be replaced with metastases, particularly in the metastatic setting of resource constraints. In clinical practice, we occasionally face difficulties in performing biopsies of metastatic sites because of tumor location or size, patient comorbidity, etc. However, we should keep in mind that because the status of ~20% of cases could still change when patients experience recurrence, the re-biopsy of a metastasis should be considered for making a precise decision. Our findings corroborate recently presented data, which showed the rate of concordance for PD-L1 staining between primaries and metastases to be 77% [21]. Since chemotherapy is an important factor changing the expression level of PD-L1, we analyzed the correlation of chemotherapy and change of PD-L1 in primary and metastasis. There is no correlation between previous chemotherapy and PD-L1 in our study.

Here, we used the SP142 antibody and considered tumors to be positive if at least 5% of cells showed membranous staining of any intensity. A concordance of nearly 80% between primary and metastatic tumor by IHC is reasonable, especially considering that various assays only have about 75% concordance, depending on how it is measured and what cut-off points and antibodies are used [16–18]. Consistent with a previous report, PD-L1 levels were higher in tumors with SCC histology than in adenocarcinomas and lower in mutant *EGFR* than in wild-type *EGFR* carriers. PD-L1 expression in biopsy specimens was recently found to be poorly correlated with that of the corresponding resected tumor in NSCLC patients [16]. Thus, in this study, we excluded biopsied samples of metastases, including core and bronchoscopic biopsies, to overcome the limitation of inter- or intratumoral heterogeneity in cases of small biopsied specimens. Our cohort of NSCLC patients had undergone surgical resection of metastatic tumor and had both primary and matched metastatic tumor samples available. We consecutively included NSCLC patients who underwent surgical resection, and thus, most had adenocarcinoma with single or few metastases and *EGFR* mutations. We reasoned that the inclusion of such patients would be a more appropriate indication for surgical resection of metastases compared to those with multiple and aggressive metastatic features. Our results showed that about 20% of primaries were PD-L1 positive and almost 70% had a score of 0, which is less than that reported earlier [11]. The aforementioned less aggressive features in our cohort might affect the low frequency of PD-L1 positivity.

We also explored the RISH method for detecting PD-L1 in primaries and metastases, and compared two different platforms—IHC and RISH—in the same patient cohort. To our knowledge, this is the first study to compare PD-L1 RISH between primaries and metastases in a cohort of surgically resected metastatic tumors in NSCLC patients. On the basis of RISH positivity scores of ≥1, about 60% concordance between primaries and metastases was observed, slightly lower than the IHC results. Approximately 70% concordance across the two different platforms was observed between primaries and metastases.

Our study had several limitations such as selection bias owing to the retrospective design, as well as small sample size. Hence, our findings should be interpreted with caution, and further studies with larger sample sizes should be performed to confirm these results. Moreover, we could not determine the response to PD-1 blockade in this trial because no patient received PD-1 blockade therapy.

In conclusion, we demonstrated that the PD-L1 status of primaries is consistent with that of metastases at recurrence in NSCLC. However, re-biopsy should be considered with caution to make correct immunotherapeutic decisions in NSCLC patients because of the inconsistency of PD-L1 status in approximately 20% of patients.

## MATERIALS AND METHODS

### Patients

This study was conducted in a cohort of NSCLC patients who underwent surgical resection at Severance Hospital in Seoul, Korea, between 2005 and 2012. The inclusion criteria were (1) surgical resection of primary NSCLC with a curative aim at initial diagnosis, (2) surgically resected metastatic lesions at recurrence, (3) availability of paired primary and metastatic tumor tissue, and (4) availability of clinical data on smoking status and survival. Paired tumor samples from 37 patients were used for examining PD-L1 expression. Tumors were classified according to the seventh American Joint Committee on Cancer (AJCC) TNM cancer classification system and the World Health Organization system. A predesigned data collection format was used to review the patients’ medical records for evaluating clinicopathological characteristics and survival outcomes. The study was approved by the Institutional Review Board of Severance Hospital.

### IHC

Sections of FFPE tissues were prepared and stained with hematoxylin and eosin. IHC was performed on 4-μm tissue sections using the Ventana Bench Mark XT Autostainer (Ventana Medical Systems, Tucson, AZ, USA) and the SP142 antibody (dilution 1:100; Ventana). PD-L1 positivity was defined as a membranous staining intensity of ≥5%. IHC scoring was done on a 0–2 scale (0 = <5%, 1 = 5%–49%, and 2 = ≥50%) [6]. The semi-quantitative H score (maximum value of 300 corresponding to 100% of tumor PD-L1-positive cells with an overall staining intensity score of 3) was determined by multiplying the percentage of stained cells by the intensity score (0, absent; 1, weak; 2, moderate; and 3, strong). Two experienced pathologists (H.S.S and Y.J.C) blinded to the patients’ clinical information examined the PD-L1 expression. For specimens with discrepant results, two pathologists re-evaluated the PD-L1 positivity status to reach a consensus after consultation.

### RISH

RISH was performed on FFPE sections using the ViewRNA^®^ eZ-L assay (Affymetrix, Santa Clara, CA, USA) on the Leica Bond III Immunohistochemistry and ISH Staining System (Leica Biosystems). Each case was assayed using probes for PD-L1. The ViewRNA eZ Check Human probe (GAPDH, ACTB, and PPIB) was used as the positive control to determine RNA integrity. The *Bacillus subtilis* probe DapB was used as the negative control to determine assay background. FFPE sections on slides were processed automatically from deparaffinization to ISH staining and hematoxylin counterstaining. Briefly, 4-μm-thick FFPE sections were baked for 1 h at 60°C and placed on the Bond III for processing. Next, slides were rinsed with water, air-dried for 30 min at room temperature, mounted using Dako Ultramount (Dako, Carpinteria, CA, USA), and visualized using a standard bright-field microscope. Punctate dot-like red hybridization signals in the cytoplasm of tumor cells indicated positive staining. RISH scoring was done on a scale of 0–3 as follows: 0, <1 dot/cell at 400× magnification; 1, 1–5 dots/cell at 400× magnification; 2, 6–20 dots/cell at 400× magnification; and 3, >20 dots/cell at 400× magnification. At least three high-power fields were analyzed before assigning an RISH score to each case.

### Statistical analysis

Correlations between immune markers and patient characteristics were analyzed using the chi-squared test with χ^2^ correction or Fisher's exact test for categorical variables. To compare dichotomized values, we calculated the proportion of discordance between both procedures together with 95% confidence intervals, as well as Cohen's κ coefficient of agreement. Survival variables were estimated using the Kaplan–Meier method. Categorical variables were compared via the log-rank test, and quantitative variables by a Cox regression model and the associated Wald chi-square statistic. Overall survival (OS) was defined as the time from the initial diagnosis until death or the most recent follow-up. Relapse-free survival (RFS) was measured from the time of surgery to initial tumor relapse (local or distant recurrence) or death from any cause. Patients with no signs of relapse were censored at the most recent follow-up or death. The median follow-up duration for the overall population was 46.3 months. A two-sided *P*-value of <0.05 was considered statistically significant. All the statistical analyses were performed using SPSS 20.0 for Windows.

## SUPPLEMENTARY MATERIALS FIGURES


