# Supplementary material for: Concordance of programmed death-ligand 1 expression between primary and metastatic non-small cell lung cancer by immunohistochemistry and RNA in situ hybridization
Source: Oncotarget. 2017 Aug 14;8(50):87234–43. doi: 10.18632/oncotarget.20254 (PMC5675629; doi:10.18632/oncotarget.20254)
Supplement: Supplementary file 1 [file oncotarget-08-87234-s001.pdf]

Concordance of programmed death-ligand 1 expression between primary and metastatic non-small cell lung cancer by immunohistochemistry and RNA *in situ* hybridization

SUPPLEMENTARY MATERIALS

	Immunohistochemistry SP142		In situ hybridization ViewRNA		
	Scoring criteria: 0 : < 5% 1 : ≥ 5% but < 50% 2: ≥ 50%		Scoring criteria: 0 = ≤1 dot/ cell 1 = 2-5 dots/cell 2 = 6-20 dots/cell		
PD-L1 expression	Primary	Metastatic	Primary	Metastatic	CASE number
	2	0	2	0	1
	2	2	2	2	2
	2	1	1	1	3
	2	0	2	0	4
	1	1	0	1	5
	1	1	0	1	6
	1	0	0	0	7
	0	1	1	1	8
	0	2	0	2	9
	0	0	0	0	10
	0	1	0	1	11
	0	0	0	0	12
	0	0	1	0	13
	0	0	1	0	14
	0	0	0	0	15
	0	0	0	0	16
	0	0	0	0	17
	0	1	1	1	18
	0	0	1	0	19
	0	0	1	0	20
	0	0	1	0	21
	0	0	1	0	22
	0	0	1	0	23
	0	0	0	0	24
	0	0	0	0	25
	0	0	0	0	26
	0	0	0	0	27
	0	0	0	0	28
	0	0	0	0	29
	0	0	0	0	30
	0	0	0	0	31
	0	0	0	0	32
	0	0	0	0	33
	0	0	0	0	34
	0	0	0	0	35
	0	0	0	0	36
	0	0	0	0	37

Supplementary Figure 1: Detailed scores of PD-L1 immunohistochemistry (IHC) and RNA *in situ* hybridization (RISH) in primary and metastatic tumor tissues in 37 patients.

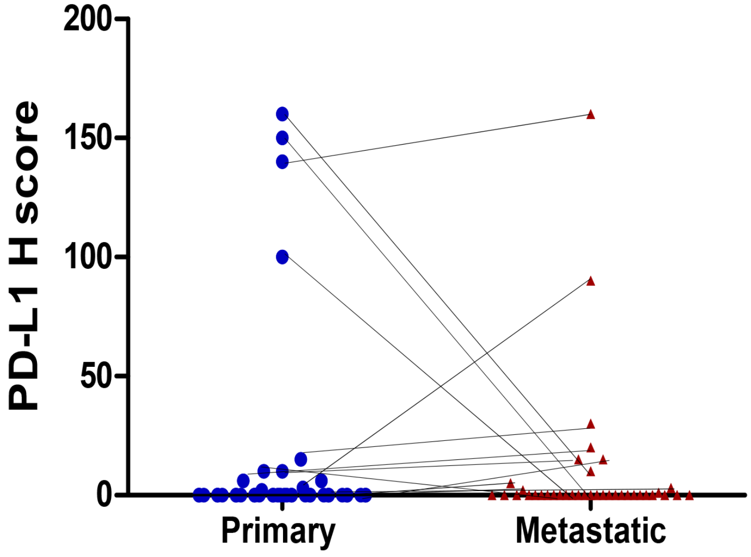

Supplementary Figure 2: The H-score of PD-L1 changes in primaries at initial diagnosis and metastases at recurrence.
